# Hereditary α-Tryptasemia and Peripheral Blood *KIT* D816V Mutation in Patients with Pediatric Mastocytosis

**DOI:** 10.3390/ijms26136023

**Published:** 2025-06-23

**Authors:** Olga Tockova, Tanja Planinsek Rucigaj, Simona Ivancan, Urska Bidovec Stojkovic, Matija Rijavec, Julij Šelb, Peter Korošec

**Affiliations:** 1 Department of Dermatovenereology, University Medical Centre Ljubljana, 1000 Ljubljana, Slovenia; olga.tockova@kclj.si (O.T.); tanja.planinsekrucigaj@kclj.si (T.P.R.); 2 Children’s Hospital, Department of Hematology and Oncology, University Medical Center Ljubljana, 1000 Ljubljana, Slovenia; simona.ivancan@kclj.si; 3 Laboratory for Clinical Immunology & Molecular Genetics, University Clinic of Respiratory and Allergic Diseases, 4204 Golnik, Slovenia; urska.bidovec-stojkovic@klinika-golnik.si (U.B.S.); matija.rijavec@klinika-golnik.si (M.R.); julij.selb@klinika-golnik.si (J.Š.); 4 Biotechnical Faculty, University of Ljubljana, 1000 Ljubljana, Slovenia; 5 Faculty of Pharmacy, University of Ljubljana, 1000 Ljubljana, Slovenia

**Keywords:** mastocytosis, *KIT* D816V, hereditary α-tryptasemia, pediatrics, diagnosis, peripheral blood, bone marrow

## Abstract

Hereditary α-tryptasemia (HαT)—a genetic trait caused by increased α-tryptase-encoding typtase alpha/beta-1 (TPSAB1) copy number—is associated with adult mastocytosis. The primary objective was to assess the association between α-tryptase and pediatric mastocytosis. We also want to evaluate whether the *KIT* p.D816V mutation in peripheral blood leukocytes (PBLs) reliably predicts systemic mastocytosis (SM) in children. A prospective cohort of 68 children from a referral center in Slovenia with cutaneous mastocytosis (CM) underwent tryptase genotyping by droplet digital PCR and examination for *KIT* p.D816V in PBL using a sensitive PCR test. A significant majority of patients (57 of 68; [83.8%]) had at least one α-tryptase-encoding gene; none had HαT. 7 of the 68 (10.3%) who were positive for *KIT* p.D816V in PBL, one fulfilled diagnostic criteria for indolent SM, and another was diagnosed with monoclonal mast cell activation syndrome. One of those individuals had an increased basal serum tryptase (BST) level (14.5 ng/mL). We found a high presence of germline α-tryptase in children with CM, but not HαT. By employing sensitive examination for *KIT* p.D816V in PBL, in combination with clinical data and other examinations, our study suggests that *KIT* p.D816V in PBL may indicate systemic disease in children with CM.

## 1. Introduction

Mastocytosis encompasses a heterogeneous group of clonal disorders characterized by abnormal expansion of mast cells (MCs) that accumulate in one or more organ system [1]. In systemic forms, the bone marrow (BM) and/or other extracutaneous tissues are affected, while the WHO classification defines cutaneous mastocytosis (CM) as mastocytosis solely present in the skin [1,2,3]. Pediatric mastocytosis is most frequently self-limiting and characterized by the accumulation of MC in the skin [1]. According to the international consensus statement, characteristic presentations of CM are maculopapular cutaneous mastocytosis (MPCM), with two variants, namely monomorphic (mMPCM) and polymorphic (pMPCM), diffuse cutaneous mastocytosis (DCM), and cutaneous mastocytoma (MS) with isolated (iMS) or multilocalized (mMS) form [4,5,6]. pMPCM is the major phenotypic variant in children [1,2,5,7,8,9,10,11,12]. It is characterized by brown to red oval lesions, plaques, and nodules of different sizes, distributed asymmetrically on the skin [1,2,3,4,5,6,7]. Nodular lesions, which may evolve into plaques, and atypical xanthelasmoid CM with yellow papules or nodules resembling xanthomas may also present skin manifestations in children [1,4]. Those clinical features must be distinguished from mastocytoma, which presents as a solitary or a maximum of three elevated brown or yellow lesions. Small brownish maculopapular lesions of identical shape and size are associated with mMPCM, while DCM patients present with generalized erythroderma, pachydermia, and blistering. Despite the fact that the clinical phenotype of childhood-onset mastocytosis is heterogeneous, and that the morphology of skin lesions may change during the course of the disease, a pathognomonic feature of all forms of CM is a positive Darier’s sign. It presents, in up to 90% of patients, as a wheal-and-flare reaction, which occurs within a few minutes after stroking a CM lesion [1,4,7]. Additionally, children with CM can have cutaneous MC mediator-related manifestations, with pruritus as the most commonly reported symptom [1,4,7,12,13].

Systemic mastocytosis (SM) is rarely diagnosed in pediatric mastocytosis cases [7,13,14,15]. The incidence of mastocytosis is estimated at five new cases per million persons annually, and systemic involvement occurs in approximately 10% of pediatric cases [1,2,3,4,5,6,7]. In the pediatric population, symptoms are usually related to mast cell mediator release and are less often connected to organ infiltration due to the accumulation of abnormal mast cells in extracutaneous tissues. Late-onset of the disease in pediatric age, monomorphic clinical form, persistence of skin lesions after puberty, organomegaly, and significant abnormalities in the complete blood count are considered to be the predictors of SM in the pediatric population [1,7,10,13,16,17,18]. Pediatric SM is present mainly in indolent form (ISM), whereas advanced pediatric SM is very rare [2,4,11]. Unlike adults, almost all children with systemic disease have skin lesions with mMPCM as the most common type of skin features [7,10,14,17].

Somatic gain-of-function point mutations within the *KIT* gene play a crucial role in developing clonal disorders. However, detecting a *KIT* mutation at codon 816 in skin tissue does not correlate with children’s CM clinical phenotype nor with the prognosis of the disease [13,16,19,20,21,22]. Furthermore, it is less frequently detected in the skin of pediatric mastocytosis than in adult SM (36% versus 90%) [1,2,23,24]. In addition to being a minor criterion for diagnosis, in recent years, identifying and quantitating the *KIT* p.D816V mutation in peripheral blood leukocytes (PBLs) became a reliable predictor of SM in adults [14,25]. A similar approach was recently taken regarding pediatric patients; the *KIT* p.D816V mutation in PBL of children was positive in the majority of patients with SM, while it was not identified in the PBL of children known to have only cutaneous disease [14].

Besides the *KIT* mutations, inherited genetic differences in tryptase genes may be associated with MC clonal disease. All known human mast cell tryptase genes are located in a compact cluster near the end of the short arm of chromosome 16. This cluster contains four paralogous genes, TPSG1 (γ alleles), TPSB2 (β2 and β3 alleles), TPSAB1 (α and β1 alleles), and TPSD1 (δ alleles). TPSAB1 and TPSB2 sites harbor alleles of biologically relevant α- and β-tryptases, which are measured and reported as serum tryptase [26,27,28]. Patients with normal TPSAB1 copy numbers may have 0, 1, or 2 copies of α-tryptase [29].

Increased copy number variants of the tryptase alpha/beta-1 (TPSAB1) gene were identified as hereditary α-tryptasemia (HαT), and this autosomal dominant genetic trait is the cause for a significant majority (approximately 90%) of individuals with elevated basal serum tryptase level (BST) in the general population [26,27,28,29]. It is found in approximately 5.7% of the general population and is associated with various clinical symptoms, though up to two-thirds of carriers may not manifest related symptoms [26]. Elevated BST, resulting from increased α-tryptase-encoding *TPSAB1* copy number, is a consistent phenotypic marker for HαT, and correct identification of HαT is critical for the accurate interpretation of serum tryptase levels in the clinical evaluation of patients [27,28].

Moreover, recent research has demonstrated that the significance extends beyond the presence of HαT; the quantity of α-tryptases also emerges as a crucial factor. Increased α-tryptase expression is linked to increased formation of mature α/β-tryptase heterotetramers that are released during mast cell degranulation and contribute to sensitization of mast cells to vibration and vascular endothelial cell permeability in vitro [26,27,28,29]. The formation of heterotetramers depends on the relative copy number of α- and β-tryptase-encoding genes.

Furthermore, HαT is associated with clonal and nonclonal MC-associated disorders and is linked to more prevalent and/or severe anaphylaxis and increased mast cell mediator-associated symptoms [29,30,31,32]. The prevalence of HαT is three times higher in adult patients with SM (12–21%) than in the general population [11,33,34]. The increased prevalence of HαT among individuals with SM has been proposed to result from the distinct properties of α/β-tryptase heterotetramers, and the prevalence of any α-tryptase-containing allele has been reported to be significantly increased among individuals with SM [11,33,34].

Although development of a *KIT* mutation is not directly related to the increased number of copies of the *TPSAB1* gene, the high prevalence of HαT in mastocytosis hints at a potential pathogenic role of germline α-tryptase-encoding *TPSAB1* copy number gains in disease development [11,34]. Thus, HαT should be considered as the trait that can modify specific clinical phenotypes, including mastocytosis [2,26,35,36,37].

It was suggested that the frequency of HαT in SM inversely correlates with the extent of involvement of hematopoiesis by the *KIT* p.D816V mutation and disease burden [11,33,34,35,36,37,38]. HαT is associated with a lower disease burden, as expressed by a lower MC infiltration in the BM and other MC-related histopathologic features [36]. Furthermore, HαT seems to be associated with a lower prevalence of cutaneous lesions in adult clonal MC patients, and it is enriched in monoclonal MC activation syndromes (MMAS) and bone marrow mastocytosis (BMM) [11]. Further, HαT was detected in almost one-third of nonclonal MC activation syndromes (nc-MCAS) in adults [34].

Tryptase gene composition has not been prospectively evaluated in a pediatric population with CM to determine whether tryptase genotype generally, or HαT, may influence pediatric mastocytosis onset, course, and outcome. The primary objectives of this study were to assess the associations between tryptase gene composition, HαT, and CM. Further, we want to confirm if highly sensitive screening for the *KIT* p.D816V mutation in PBL is a reliable predictor of SM in children. We performed analyses in a prospective cohort of patients referred in 2016–2024 to a referral center in Slovenia. All subjects who chose to participate were tryptase-genotyped in a blinded fashion.

## 2. Results

### 2.1. Study Population

From 2016 to 2024, we newly diagnosed, clinically evaluated, and treated 68 mastocytosis patients aged less than 18 years (Table 1). The recruitment for tryptase genotyping took place from October 2022 to April 2024, as HαT was discovered in 2016 [38], and the first publication concerning HαT and mastocytosis just became available in 2021 [39,40]. The classification of mastocytoses was based on the WHO 2022 classification, which updated the consensus criteria [2,4,5,6]. MPCM was diagnosed in 47.1% of our patients (*n* = 32 of 68), which was almost less than half its usual appearance in patients under the age of 18 years (i.e., between 70 and 90 percent) [1,7,8,9]. The most common MPCM variant was pMPCM, found in 87.5% (*n* = 28 of 32). Among 32 patients with MPCM, 12.5% (*n* = 4 of 32) had mMPCM, similarly as reported in the literature [7,8,9]. MS was found in 52.9% of patients (*n* = 36 of 68), presenting CM’s most common clinical presentation in our cohort. This prevalence was more than 4-fold higher than commonly described 10 to 15 percent of children with CM [1,7,8,9,13,14,19]. Among them, the isolated form was diagnosed in the majority of cases *(n*= 33 of 36 [91.7%]), and three patients (*n* = 3 of 36 [8.3%]) had mMS. DCM was not diagnosed in our CM cohort.

We had 46 male and 22 female patients with CM. The male-to-female ratio was 2.1:1, which confirmed previously reported male predominance in pediatric mastocytosis [1,9]. Only 7.4% of patients (*n* = 5 of 68) had a positive family history of mastocytosis. Our data confirmed that CM should generally not be considered a hereditary disease [7,10,11,19].

The median age at the onset of CM was 4 months (IQR 1.3–12), corresponding with previous data from the literature [1,12]. Congenital mastocytosis was diagnosed in 16.2% of patients (*n* = 11 of 68), with MS as the only clinical feature. One-third of patients with MS (*n* = 11 of 36; [30.5%]) presented with a congenital form; this is 2-fold less than previously reported [1,12]. Approximately one-fifth of our cohort still had skin changes at age 12 and over (*n* = 14 of 68; [20.5%]). This was in line with the expected spontaneous regression of CM in up to 80% of patients until puberty [1,7,10,12,13]. Interestingly, all our adolescents had clinical features of pMPCM and iMS, but not mMPCM, which usually has a higher tendency to persist into adulthood [1,9,10,11].

Although 3-fold less than in previous reports, pruritus was the most common cutaneous MC mediator-related symptom [1,4,13,16]. It was found in 10 patients (14.7%). Flushing appeared in only 8.8% of patients (*n* = 4 of 68), which was nearly 6-fold less than expected (i.e., between 30 and 50% of patients) [7,8,16]. Four children (5.8%) had a medical history of acute urticaria, and one patient (1.5%) occasionally showed bullous changes on the skin. Six patients (8.8%) had digestive symptoms, five had abdominal pain (*n* = 5 of 68 [7.4%]), and one had diarrhea (1.5%). Reflux was not present.

None of our patients had massive mast cell activation symptoms or adverse drug or vaccine reactions. Further, we documented no history of anaphylaxis to insect venom, food, drugs, or idiopathic—similarly, anaphylaxis was previously described only in approximately 4% of children with mastocytosis [1,13,14,16]. The presence of allergic disorders (*n* = 8 of 68; [1.8%]) was lower than in previous studies (i.e., between 20 and 30 percent) [23,35]. One patient with pMPCM had asthma (1.5%), four patients had pollen (5.9%), three food allergies (4%), and three patients had atopic dermatitis (4%). No other comorbidities were reported.

### 2.2. The Majority of Children with CM Have at Least One α-Tryptase-Encoding Gene Copy; However, None of Them Have HαT

**Figure 1:**
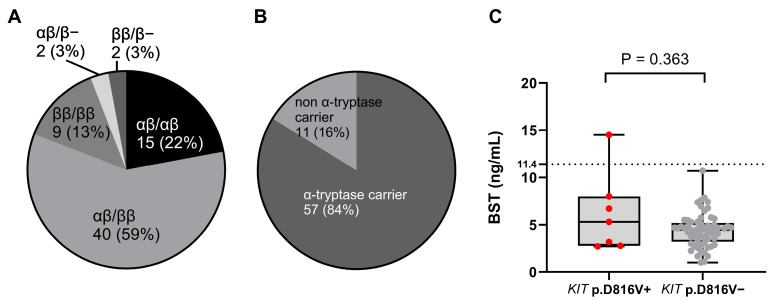
(**A**) The distribution of tryptase genotypes and (**B**) the prevalence of carriers with α-tryptase in patients with pediatric mastocytosis. (**C**) The relationship between BST levels and the presence of *KIT* p.D816V. The data are presented as boxes and whiskers. The horizontal line within the box represents the median and the box represents the 25th to 75th percentile. The whiskers represent the ranges. *KIT* p.D816V+ patients are represented by red dots and *KIT* p.D816V− by grey dots. Mann–Whitney test was used for calculation. BST: basal serum tryptase.

Of the 68 subjects included in the CM cohort, none of those individuals had HαT. However, most subjects (*n* =57 of 68; [83.8%]) had at least one α-tryptase-encoding gene; this is a much higher frequency than expected in the general population (68%) (Figure 1) [3,41].

**Figure 2:**
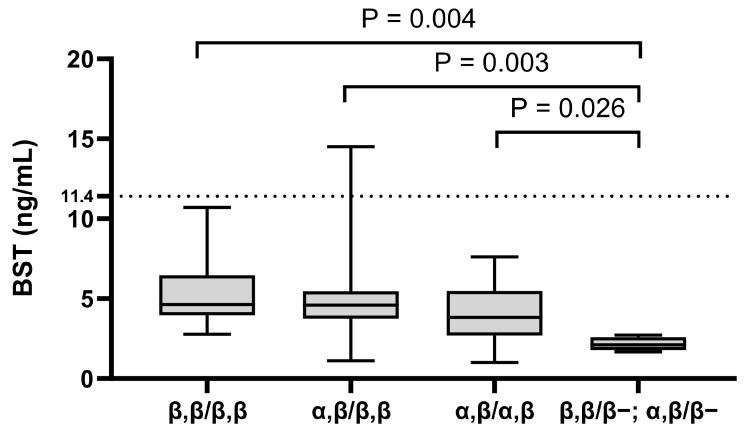
The relationship between BST levels and tryptase genotype. The data are presented as boxes and whiskers. The horizontal line within the box represents the median and the box represents the 25th to 75th percentile. The whiskers represent the ranges. Kruskal–Wallis test followed by Dunn’s post hoc test was used for calculation. BST: basal serum tryptase.

Interestingly, all patients (*n* = 10 of 10; [100%]) with pruritus were α-tryptase carriers. Of the 68 subjects, only 16.1% (*n* = 11) were genotyped as ββ:ββ or 0β:ββ (Figure 1). This frequency was 100% lower than the expected prevalence of β genotypes in the general population (32%) [40,41]. The most common tryptase genotype was αβ:ββ (*n* = 40 of 68; [58.8%]), which is also the most common genotype in the general population (Figure 1) (Table 2) [28,29,41,42]. Additionally, analysis by tryptase genotype showed that patients with the deletion had significantly lower BST levels, with a median of only 2.1 ng/mL, than patients with other tryptase genotypes, which showed no significant differences (Figure 2).

### 2.3. KIT p.D816V in PBL Represents Reliable Predictor of Systemic Clonal Disorder in Children with CM

**Figure 3:**
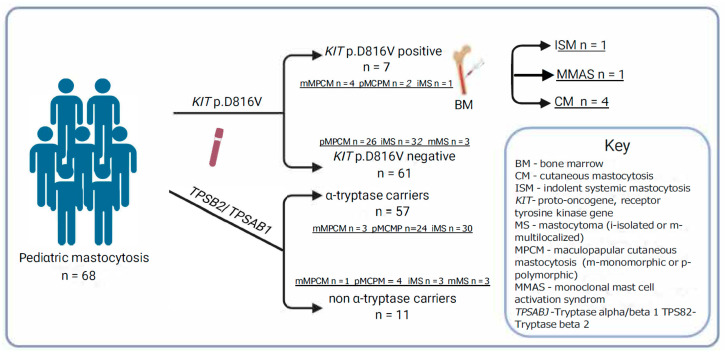
Mastocytosis subtypes according to the presence of the KIT p.D816V mutation in peripheral blood leukocytes, α-tryptase-encoding gene, and bone marrow studies.

A total of 7 of 68 patients (10.3%) were positive for *KIT* p.D816V in PBL. The median *KIT* p.D816V variant allele frequency in PBL was 0.007% (0.001–0.801%) (Table 3), and thus the majority of *KIT*-positive patients have a very low allele burden in PBL (four of six (67%): ≤0.01%). Six of seven *KIT*+ patients (85.7%) had clinical features of MPCM and one had iMS (Figure 3).

All four patients with mMPCM had *KIT* p.D816V in PBL (*n* = 4 of 6; [66.7%]; *p* = 0.002). Those findings demonstrate that in our cohort, *KIT* p.D816V in PBL was associated with MPCM, especially in monomorphic form (Figure 3). Further, pruritus was significantly more common in *KIT* p.D816V + patients compared to *KIT* p.D816V individuals (57.1% versus 9.8%; *p* = 0.03). Those observations suggest that pruritus may be an MC activation symptom in CM children with *KIT* mutation in PBL. Median levels of BST between *KIT* p.D816V + and *KIT* p.D816V patients were comparable [median (IQR) 5.3 (2.8–8.0) vs. median 4.5 (3.3–5.2), retrospectively] (Figure 1).

According to the proposed diagnostic algorithm for patients with the *KIT* p.D816V mutation in PBL, we performed a bone marrow biopsy [1,2,6,7,8,14,17,31,43,44,45] (Table 3). It was performed on six patients because we could not obtain parental consent for the seventh patient. As the diagnostic criteria of SM in children and adults are the same, the classification was based on the WHO 2022 updated consensus criteria [1,5,6] (Table S1). One patient with mMPCM (14.3%) had spindle-shaped MC infiltrates detected in sections of BM and CD2/CD25 expression. Thereby, three minor diagnostic criteria were fulfilled, and a diagnosis of ISM was established (Figure 3) [1,2,3,5,6,7,10,11]. These findings were comparable with previous observations that ISM is the most common SM in children, and mMPCM is the most common skin finding in SM [1,7,14,15,16]. The second patient with mMPCM fulfilled two minor criteria, *KIT* p.D816V in PBL and CD25/CD2 expression, and thus monoclonal mast cell activation syndrome (MMAS) was diagnosed (Figure 3). For the other four patients, no bone marrow changes were found.

Our findings demonstrate that some children with CM may develop SM, preferably those with *KIT* p.D816V in PBL. Therefore, some *KIT*-positive CM patients may require a BM biopsy and/or further laboratory testing. However, *KIT* screening in PBL should be sensitive enough, as the majority of *KIT*-positive patients had a low allele burden in PBL, and it was similar in other studies [23,25].

### 2.4. BST Level Was Increased in Only One Patient

Of 68 CM patients, 1 patient (1.5%) had an elevated BST level (14.5 ng/mL; >11.4 ng/mL). Furthermore, the patient with elevated BST levels (14.5 ng/mL) tested positive for *KIT* p.D816V in PBL (Figure 1) and was diagnosed with ISM after bone marrow analysis. There were eight patients (*n* = 8 of 68; [11.8%]) with BST levels higher than 6.5 ng/mL, a value with the possibility for HαT in children. The BST levels higher than 6.5 ng/mL are not highly indicative of HαT, but rather, in patients with BST levels lower than 6.5 ng/mL, the HαT is highly unlikely, since no individual has been reported or observed with HαT and a BST level less than 6 ng/mL [28]. 

The median BST level in our cohort was 4.6 ng/mL (IQR 3.2–5.3) (Table 1). That is comparable to the median value of BST in the general population (5 ng/mL) [1,34,46]. However, we had no patients with DCM nor an aggressive form of SM—those forms are most often associated with elevated BST in children [1,3,7,30,47]. It is worth mentioning that in CM patients with detectable *KIT* p.D816V in PBL, the median BST levels (4.3 ng/mL) were highly comparable to those of the whole cohort. Those observations suggest that *KIT* screening may identify systemic disease in patients with CM otherwise missed using BST and organomegaly alone.

We also performed a multivariate linear regression analysis to determine whether there is a significant association between BST and the tryptase genotype, *KIT* p.D816V, and major clinical variables presented in Figure 1 and Figure 2 and Table 4. The analysis showed that only the presence of cutaneous symptoms is a significant positive predictor of BST; there was also a positive trend for the *KIT* p.D816V mutation (*p* = 0.053).

## 3. Discussion

Our study demonstrates that the presence of germline α-tryptase-encoding sequences is increased in patients with CM. However, we could not confirm that CM is associated with HαT, which was previously shown for SM. Further, we showed that *KIT* p.D816V screening in PBL is essential to identify the subgroup of children with CM at risk of SM.

According to the established connection between the higher frequency of HαT and lower disease burden in mastocytosis, Zama et al. recently published the first pediatric case of HαT in patients with clinical manifestation of MS [48]. Madrange et al. similarly show that in pediatric mastocytosis, HαT seems to be associated with mastocytoma [49]. Although more than half of our cohort had MS, our study could not confirm the association between HαT and mastocytoma. Overall, our observations suggest a much lower prevalence of HαT in pediatric-onset mastocytosis than in adults. Whereas none of our CM children have HαT, we demonstrated a markedly higher prevalence of germline α-tryptase-encoding sequences in our CM cohort than in the general population (84% versus 65%) [3,50,51]. Furthermore, all patients with pruritus (*n* = 10; [100.0%]) were α-tryptase carriers. These findings suggest that α-tryptase, not HαT, may generally be associated with pediatric mastocytosis and MC mediator-related symptoms in CM patients.

A recent study demonstrates that the presence of germline α tryptase-encoding sequences is associated with more severe allergic reactions to foods [52]. Thus, alpha/β-tryptase heterotetramers, produced if an individual carries the α-tryptase gene, may contribute to the pathogenesis of MC-mediated reactions [40]. It is worth mentioning that the presence of α-tryptase was strongly enriched in our CM subjects with pruritus, which further suggests the possible importance of heterotetramers in MC-related symptoms.

As the *KIT* p.D816V mutation in PBL became a reliable predictor of SM in adults, Carter et al. suggested that *KIT* p.D816V mutation in PBL may also indicate systemic disease in children [17]. In a study of 32 Polish children with CM, a *KIT* p.D816V mutation was detected in the PBL in 34% of patients, and four of five *KIT*-positive individuals undergoing bone marrow biopsy showed BM changes [45]. Our study found the *KIT* p.D816V mutation in PBL of 10.3% of patients with CM. Notably, all four children with mMPCM and two with pMPCM were *KIT*-positive. Further, following BM study, systemic clonal MC disorder was diagnosed in the mMPCM patient. Based on those observations and previous data, MPCM remains the key clinical sign that leads to the diagnosis of systemic MC disorder in children, although pMPCM is mainly present as a skin-limited disease [1,7,8,9,10,13,14]. Additionally, pruritus was significantly more common in *KIT* p.D816V-positive individuals, suggesting that the *KIT* mutation may affect MC activation symptoms in CM children.

The relationship between the *KIT* D816V mutation and clinical manifestations might also be affected by confounding factors such as patient age, gender, and allele burden. For example, Broesby-Olsen et al. [53] conducted a study involving 48 adult patients with ISM to evaluate the influence of the level of *KIT* D816V-positive cells in peripheral blood or bone marrow and found that patients with the highest mutation burden tended to be older and had longer disease durations. Hoermann et al. [54] assessed the *KIT* D816V allele burden in 105 patients with systemic mastocytosis, revealing significant differences in allele burdens across disease subtypes and prognostic significance concerning survival. However, we could not analyze those confounding factors in detail due to our study’s limited number of *KIT* D816V-positive individuals (*n* = 7). Nevertheless, of our seven *KIT*-positive patients, five children with low (<0.05%) allelic burden tend to be younger (5y, 5y, 7y, 8y, and 11y) than two children with moderate (>0.3%) burden (9y and 11y; see Table 3). Further, only the child with the highest (0.8%) burden of those seven individuals was diagnosed with systemic mastocytosis (ISM).

We also found the *KIT* p.D816V mutation in one patient with iMS. According to the previous data, the *KIT* mutation is very rarely seen in iMS patients, and overall, only 10 iMS patients were described with *KIT* p.D816V in the skin bioptat [23,26,55,56]. Our study is the first to find *KIT* p.D816V in PBL of iMS patients. This finding suggests that PBL *KIT* screening and careful clinical evaluation should be carried out on all children with CM, as any skin manifestations of CM may be related to *KIT* mutations and underlying systemic disorders.

iMS was the most frequent clinical variant of CM (38.5%) in our study. Until now, only Hannaford et al. reported the occurrence of iMS in 51% of CM patients; otherwise, it usually accounts for 10 to 15% of pediatric cases of CM [1,7,57]. Generally, MS is exceptionally rarely associated with SM [1,7,8,13]. Chantorn et al. reported progression to SM in a patient who had MS since the age of five [58]. Interestingly, one of our patients had MS since they were five years old. Subsequently, we should be aware that such cases need close monitoring. A case of malignant transformation of MS to mast cell sarcoma in a 40-year-old man with a persistent neonatal MS was reported [18]. Accordingly, we should emphasize the need for close follow-up of patients with congenital MS [1,7,11,12,13,16].

In our analysis, we have used three different cut-off values of BST. The lower detection limit of BST measured using a commercially available fluorescence enzyme ImmunoCAP immunoassay is 1 ng/mL, and the normal range for total tryptase levels in serum ranged from 1 to 11.4 ng/mL [32]. In our patient with elevated BST levels (14.5 ng/mL) who tested positive for *KIT* pD816V in PBL and was diagnosed with ISM after bone marrow analysis, we used BST cut-off of >20 ng/mL, which is one of the diagnostic criteria of SM (see Table S1) and which was not reached in this patient. Further, if there is an associated myeloid neoplasia, this criterion is not valid, and it should be further adjusted in case of HαT [1,5,6]. Finally, there were also eight patients (*n* = 8 of 68; [11.8%]) with BST levels higher than 6.5 ng/mL, a value with the possibility for HαT in children. In the case of BST levels lower than 6.5 ng/mL, the HαT is highly unlikely since no individual has been reported or observed with HαT and a BST level less than 6 ng/mL [28]. It is also worth mentioning that the lowest BST levels (approximately 2 ng/mL) were found in patients with the deletion in the tryptase genotype; this is a new observation and should be further explored.

Finally, we want to point out that the potential limitation of our study might be the relatively small sample size, which may limit the statistical power and generalizability of the findings. The relatively small sample resulted from the strict inclusion criteria in the only referral center for CM in Slovenia and the CM epidemiology in a state with a population of 2 million. In addition, in relation to the number of patients, the incidence of CM is approximately 5 new patients per 1 million [1,4]. As we are a nation of 2 million, we should have ca 80 patients from 2016 to 2024, and we had 68 patients. This is very close to 80, and we should include cohorts from other larger countries to significantly increase the number of patients. Nevertheless, further research involving larger, multicenter cohorts will be essential to validate our results and explore additional associations that could not be examined in the current dataset.

While the current study employed conventional statistical methods tailored to small sample sizes, we acknowledge that more advanced analytical approaches, such as machine learning algorithms or unsupervised clustering techniques, may offer further insights into complex patterns within the data. However, given our cohort’s limited number of participants, applying such models would carry a high risk of overfitting and limited interpretability. We, therefore, opted for statistically robust non-parametric methods to ensure reliable results. Future studies with larger datasets may benefit from incorporating machine learning frameworks to explore predictive modeling or subgroup identification in greater depth.

## 4. Materials and Methods

### 4.1. Study Cohort

We prospectively recruited 68 newly diagnosed patients, ages 6 months to 17 years, with CM referred to the Clinic of Dermatovenereology University Medical Centre Ljubljana from January 2016 to March 2024 (Table 1). The recruitment for the tryptase genotyping took place from October 2022 to April 2024. Our center is the only referral center for children with CM in Slovenia. None of the patients referred to our center underwent prior screening; thus, they were not selected for clonal MC disease before evaluation. Complete history and physical examination were performed, and patients were classified based on clinical presentation and according to the current WHO classification of mastocytosis [2,3]. The diagnosis of CM was established according to the proposed criteria, which were defined by clinical presentation and a positive Darier’s sign as primary criteria [1,2,3,4,5]. Considering the international consensus statement, characteristic presentations of childhood CM in MPCM and MS were diagnosed [4,5,6,7,8]. Additionally, we recognized monomorphic and polymorphic variants of MPCM and two clinical variants of MS, iMS, and mMS. DCM was not diagnosed.

According to the criteria, skin biopsy, with histopathological examination and immunophenotyping, is required only in cases with atypical or non-diagnostic CM, negative Darier’s sign, and in cases where other dermatological diseases are included in the differential diagnosis [1,4,5]. We had no clinically unclear cases; subsequently, there was no need for additional workup and histopathological verification of the diagnosis [7,8].

Patients’ age at the onset of the disease, mastocytosis type, presence of MC activation symptoms, allergic and other comorbidities, gender, and personal and family history were also evaluated. Determination of BST levels and tryptase genotyping studies were obtained for all study participants. Likewise, in all subjects, the activating *KIT* c.2447A > T, p.D816V missense variant, and allele burden were assayed in PBL. Ethical approval was obtained from the Slovenian National Medical Ethics Committee (0120-221/2022/8). All subjects and their parents/guardians provided written informed consent.

### 4.2. Total Serum Tryptase

BST levels were measured using a commercially available fluorescence enzyme ImmunoCAP immunoassay (Thermo Fisher Scientific, Uppsala, Sweden). The lower detection limit was 1 ng/mL, and the normal range for total tryptase levels in serum ranged from 1 to 11.4 ng/mL [32].

### 4.3. KIT p.D816V in PBL

Genomic DNA was extracted from 400 µL of EDTA-containing whole blood samples using a QIAamp DNA Mini Kit (Qiagen, Hilden, Germany) on a fully automated QIACube System (Qiagen) according to the manufacturer’s instructions. The activating *KIT* c.2447A > T, p.D816V missense variant, and allele burden were assayed by allele-highly sensitive quantitative PCR (qPCR) using the ABI 7500 Fast Real-Time PCR system and SDS 2.3 software (Thermo Fisher Scientific) [30,41,43,59,60]. All qPCR runs included a no-template control (water), p.D816V-negative control, and p.D816V-positive control sample.

### 4.4. Tryptase Genotyping

*TPSAB1* and *TPSB2* genotyping was accomplished by multiplex droplet digital PCR (ddPCR) assay, as described recently [28], on all CM individuals independently of BST levels. *TPSAB1* and *TPSB2* copy numbers were assessed using custom primers and probes specifically targeting α- and β-tryptase sequences together with primers and probes targeting *AP3B1* or *AGO1* as reference genes using a manual droplet generator (Bio-Rad, Hercules, CA, USA), QX200 droplet reader (Bio-Rad), and associated QX Manager software 2.0 (Bio-Rad) [28,30,43,44,51,60]. All ddPCR runs included a no-template control (water) and a control sample with known *TPSAB1* and *TPSB2* copy numbers.

### 4.5. Bone Marrow Studies

Bone marrow aspiration and bone marrow biopsy were performed under general anesthesia according to the institutional SOP (ND 4096 and ND 4083; Children’s Hospital, Department of Hematology and Oncology, University Medical Center Ljubljana) and international recommendations [10,38,61,62]. The flow cytometry of bone marrow aspirate was subsequently performed according to standard protocols for systemic mastocytosis [42]. The bone marrow aspirates were stained with antibodies specific for CD2, CD25, CD45, and CD117 [45].

### 4.6. Statistical Analysis

GraphPad Prism 10 software (version 10.2.1 for Windows; GraphPad Software, San Diego, CA, USA) and R 4.4.3 Statistical Computing software (R Foundation for Statistical Computing, Vienna, Austria) were used for statistical analysis. Descriptive statistics included frequencies and proportions for categorical variables; median and interquartile ranges (IQR) for non-normally distributed numerical variables. A *p*-value < 0.05 was considered statistically significant. Categorical variables were assessed with Fisher’s exact test. Numerical variables with normal distribution and those not normally distributed were analyzed using Student’s *T* test, Mann–Whitney test, and Kruskal–Wallis test, followed by Dunn’s post hoc test, respectively; in all cases, two-tailed tests were used. We performed a multivariate linear regression analysis to determine whether there is a significant association between BST (the dependent variable) and the other independent variables/predictors. The coefficients are beta values of the linear regression model, and *p*-values represent the levels of significance of these coefficients.

## 5. Conclusions

We found a high presence of germline α-tryptase in children with CM and showed that all CM children with pruritus have α-tryptase sequences. However, none of the CM individuals have HαT. These findings expand our understanding of MC-related disorders in CM children. Further, by employing universal and sensitive examination for *KIT* p.D816V in PBL, we highlight the potency of *KIT* screening in PBL to find CM patients at risk for systemic clonal MC disorder and further highlight the use of *KIT* screening as a biomarker in clinical practice of children with CM. Additional data incorporating tryptase genotyping in more extensive studies of pediatric patients with mastocytosis are needed to confirm those observations.

## Figures and Tables

**Table 1:** Main characteristics of study population.

Cohorts/Triggers /Severity	Total (*n* = 68)	KIT D816V^+^ (*n* = 7)	KIT D816V^−^ (*n* = 61)
**Sex, *n* (%)**			
Female	22 (32)	2 (29)	20 (33)
Male	46 (68)	5 (71)	41 (67)
**Age at onset [months], median (IQR)** *	4 (10.75)	2 (2.0)	6 (12.5)
**Age at clinical data [months], median (IQR)** **‡**	78 (69.0)	96 (36.0)	72 (84.0)
**Congenital mastocytosis, *n* (%)**	16.2 (11/68)	14.3 (1/7)	16.4 (10/61)
**Familial mastocytosis, *n* (%)**	7.4 (5/68)	0 (0/7)	8.2 (5/61)
**Mastocytosis subtypes (WHO classification), *n* (%)**			
Cutaneous mastocytosis	97.1 (66/68)	71.4 (5/7)	100 (61/61)
Maculopapular cutaneous mastocytosis (MPCM)	47.1 (32/68)	85.7 (6/7)	42.6 (26/61)
Monomorphic MPCM	12.5 (4/32)	66.7 (4/6)	0 (0/61)
Polymorphic MPCM	87.5 (28/32)	33.3 (2/6)	42.6(26/61)
Mastocytoma (MS)	52.9 (36/68)	14.3 (1/7)	57.4 (35/61)
Isolated MS	91.7(33/36)	100 (1/1)	91.4 (32/35)
Multilocalized MS	8.3(3/36)	0 (0/1)	8.6 (3/35)
Diffuse cutaneous mastocytosis (DCM)	0 (0/68)	0 (0/7)	0 (0/61)
Systemic mastocytosis	1.5 (1/68)	14.3 (1/7)	0 (0/61)
Indolent systemic mastocytosis	1.5 (1/68)	14.3 (1/7)	0 (0/61)
Aggressive systemic mastocytosis	0 (0/68)	0 (0/7)	0 (0/61)
Monoclonal mast cell activation (MMAS)	1.5(1/68)	14.3 (1/7)	0 (0/61)
**Mast cell activation symptoms, *n* (%)**			
Anaphylactic reactions	0 (0/68)	0 (0/7)	0 (0/61)
Cutaneous symptoms	26.5 (18/68)	57.1 (4/7)	26.2 (16/61)
Flush	8.8 (4/68)	14.3 (1/7)	4.9 (3/61)
Urticaria	5.9 (4/68)	0 (0/7)	6.5 (4/61)
Pruritus	14.7 (10/68)	57.1 (4/7)	9.8(6/61)
Bullous	1.5 (1/68)	0 (0/7)	1.6 (1/61)
Digestive symptoms	8.8 (5/68)	14.3 (1/7)	6.5 (4/61)
Abdominal pain	7.4 (5/68)	14.3 (1/7)	6.5 (4/61)
Diarrhea	1.5(1/68)	0 (0/7)	2 (1/61)
Reflux	0 (0/68)	0 (0/7)	0 (0/61)
**Allergic disorders, *n* (%)**	11.8 (8/68)	14.3 (1/7)	11.5 (7/61)
Asthma bronchial	1.5(1/68)	0 (0/7)	1.6 (1/61)
Pollen allergy	5.9 (4/68)	0 (0/7)	6.5 (4/61)
Food allergy	4.4 (3/68)	14.3 (1/7)	3.3 (2/61)
Atopic dermatitis	4.4 (3/68)	0 (0/7)	3.3 (2/61)
**Absence of mast cell activation syndrome, *n* (%)**	70.6 (48/68)	28.6 (2/7)	75.4 (46/61)
**Laboratory data**			
Tryptase [ng/mL], median (IQR)	4.6 (2.13)	6.2 (5.22)	4.5 (1.91)
Mastocytoma (*n* = 36)	4.2(1.73)	0.0 (0.0)	4.4 (1.61)
MPCM	4.7 (3.58)	6.0 (6.55)	4.6 (2.38)
Monomorphic MPCM (*n* = 4)	6.7 (9.17)	6.7 (9.17)	N/A
Polymorphic MPCM (*n* = 28)	4.6 (3.11)	4.7 (3.92)	4.6 (2.38)
DCM (*n* = 0)	0% (0/68)	0% (0/7)	0% (0/61)
Hereditary alpha-tryptasemia (HAT) (*n* = 0)	0% (0/68)	0% (0/7)	0% (0/61)

N/A, not applicable. * Age at the onset of initial symptoms before patients first came to the clinic. ‡ Age when the patient first came to the clinic, was newly diagnosed with CM, and clinically evaluated for the presented data.

**Table 2:** Tryptase genotypes.

Genotype, *n* (%)	αβ:αβ	αβ:ββ	ββ:ββ	αβ:0β	0β:ββ	HαT *	α-Tryptase Carriers	Non α-Tryptase Carriers
CM cohort (*n* = 68)	15 (22.1)	40 (58.8)	9 (13.3)	2 (2.9)	2 (2.9)	0	57 (83.8)	11 (16.1)
mMPCM (*n* = 4)	0 (0.0)	3 (75.0)	1 (25.0)	0 (0.0)	0 (0.0)	0	3 (75.0)	1 (25.0)
pMPCM (*n* = 28)	8 (28.6)	17 (60.7)	3 (10.7)	0 (0.0)	0 (0.0)	0	24 (85.7)	4 (14.3)
Mastocytoma (*n* = 36)	7 (19.4)	21 (58.3)	5 (13.9)	2 (5.6)	1 (2.8)	0	30 (83.3)	6 (16.7)
Pruritus (*n* = 10)	4 (40.0)	6 (60.0)	0 (0.0)	0 (0.0)	0 (0.0)	0	10 (100.0)	0 (0.0)
KIT D816V+ (*n* = 7)	0 (0.0)	6 (85.7)	1 (14.3)	0 (0.0)	0 (0.0)	0	6 (85.7)	1 (14.3)
KIT D816V− (*n* = 61)	15 (24.6)	34 (55.7)	8 (13.1)	2 (3.3)	2 (3.3)	0	51 (83.6)	10 (16.4)
BST >6.5 ng/mL (*n* = 9)	2 (2.9)	6 (8.8)	1 (1.6)	0 (0.0)	0 (0.0)	0	8 (88.8)	1 (11.1)
BST > 6.5 ng/mL, median	7 (0.7)	7 (0.7)	7 (4.5)	9 (2.7)	0 (0.0)	0	7 (1.0)	9 (2.7)
BST < 6.5 ng/mL (*n* = 59)	13 (19.1)	35 (51.5)	7 (10.3)	2 (3.3)	2 (3.3)	0	49 (83.1)	10 (16.9)
BST < 6.5 ng/mL, median	3 (2.1)	4 (1.4)	5 (1.3)	2 (1.1)	2 (0.2)	0	4 (1.9)	4 (2.2)

* Genotypes consistent with HαT (at least 2 copies of α-tryptase on the same allele).

**Table 3:** Bone marrow studies in individuals with detectable *KIT* p.D816V in blood leukocytes.

Sex	Age	Variant CM	BST [ng/mL]	D816V Frequency	Major	Minor a	Minor b	Minor c	Minor d	Celularity BM	Diagnosis
M	7	pMPCM	2.78	0.001%	0	0	yes	0	N	normal	CM
M	9	mMPCM	14.50	0.801%	0	yes	yes	yes	N	normal	ISM
M	11	pMPCM	6.70	0.311%	0	0	yes	0	N	normal	CM
M	11	mMPCM	3.18	0.003%	0	0	yes	yes	N	normal	MMAS
F	5	iMS	2.71	0.001%	0	0	yes	0	N	normal	CM
F	5	mMPCM	5.31	0.010%	0	0	yes	0	N	normal	CM
M *	8	mMPCM	8.00	0.048%	-	-	yes	-	-	-	CM

Major: major criterion; Minor: minor criterion; a: atypical morphology (25%); b: *KIT* point variant in B.M./blood/other organ; **c**: CD25 CD2 expression; d: BST > 20 (see Table S1). B.M.: bone marrow; S.M.: systemic mastocytosis; ISM: indolent S.M.; MMAS: monoclonal mast cell activation syndrom. Cutaneous mastocytosis (CM); MPCM: maculopapular cutaneous mastocytosis; monomorphic MPCM (mMPCM); polymorphic MPCM (pMPCM); mastocytoma of skin (MS); isolated MS (iMS); multilocated MS (mMS). * not available for bone marrow studies.

**Table 4:** Multivariate linear regression analysis of factors associated with BST levels.

Variable	Coefficient	Std. Error	*T Value*	*p*-Value
Intercept	6.127	5.403	1.134	0.2618
Age at onset	−0.053	0.057	−0.928	0.358
Sex	0.868	0.604	1.435	0.157
α-tryptase carriers	0.262	1.047	0.250	0.804
Number of α encoding gene copy	−1.020	1.370	−0.745	0.460
Tryptase genotypes	−0.359	1.095	−0.328	0.744
Deletion in the tryptase genotype	−3.987	3.492	−1.142	0.259
*KIT* p.D816V	1.715	0.869	1.974	0.053
Congenital mastocytosis	0.023	0.578	0.040	0.323
Familial mastocytosis	1.152	0.993	1.160	0.251
Cutaneous mastocytosis subtype	0.023	0.578	0.040	0.968
Cutaneous symptoms	1.997	0.573	3.486	0.001
Digestive symptoms	−1.439	1.056	−1.362	0.179
Allergic disorders	1.254	0.750	1.671	0.101

## Data Availability

The data supporting this study’s findings are available from the corresponding author upon request.

## Supplementary Materials

The following supporting information can be downloaded at: https://www.mdpi.com/article/10.3390/ijms26136023/s1.

## Abbreviations

hereditary α-tryptasemia (HαT), cutaneous mastocytosis (CM), peripheral blood leukocytes (PBLs), maculopapular cutaneous mastocytosis (MPCM), cutaneous mastocytoma (MS), monomorphic MPCM (mMPC), polymorphic (pMPCM), isolated cutaneous mastocytoma (iMS), multilocalized cutaneous mastocytoma (mMS), basal tryptase level (BST).
