# Supplementary material for: Hereditary α-Tryptasemia and Peripheral Blood KIT D816V Mutation in Patients with Pediatric Mastocytosis
Source: Int J Mol Sci. 2025 Jun 23;26(13):6023. doi: 10.3390/ijms26136023 (PMC12249517; doi:10.3390/ijms26136023)
Supplement: Supplementary file 1 [file ijms-26-06023-s001.zip › ijms-3612663-supplementary.pdf]

Table S1. Criteria for diagnosis and classification in SM[1-3].

Major criterion:
Multifocal, dense infiltrates of MC (15 or more MC in aggregates) detected in section of B.M. and/or other extracutaneous organ(s) and confirmed by tryptase immunohistochemistry or other special stains
Minor criteria:
a) In biopsy sections of BM or other extracutaneous organs, more than 25% of MC in the infiltrate are spindle-shaped or have atypical morphology, or, of all MC in BM aspirate smears, more than 25% are immature or atypical MC.
b) Detection of any activating *KIT* point mutation in BM, blood, or other extracutaneous organ(s)
c) Expression of CD25 and/or CD2 and/or CD30 on MC in BM, blood or other extracutaneous organ(s)
d) Serum total tryptase *persistently > 20 ng/ml (if there is an associated myeloid neoplasia, this criterion isn't valid)
*should be adjusted in case of hereditary alpha-tryptasaemia
B findings:
1) > 30% infiltration of cellularity by MC (focal, dense aggregates) in BM biopsy and serum total tryptase >200 ng/ml
2) Myeloproliferation or signs of dysplasia, but criteria are not met for definitive diagnosis of associated hematological neoplasms, no prominent cytopenias;
3) Hepatomegaly and/or splenomegaly on palpation without impairment of organ function and/or lymphadenopathy on palpation/imaging (> 2 cm)
4) *KIT* p.D816V mutation with Variant allele frequency (VAF)≥ 10
C findings:
1) BM dysfunctions caused by neoplastic MC infiltration manifested by one or more cytopenia: ANC < 1 x 10^9/L, Hb < 10 g/dL, or platelets < 100 x 10^9/L without other hematological neoplasms
2) Hepatomegaly on palpation with impairment of liver function, ascites, and/or portal hypertension
3) Skeletal lesions: osteolysis and/or pathologic fractures
4) Palpable splenomegaly with hypersplenism
5) Malabsorption with weight loss from gastrointestinal tract MC infiltrates

MC: mast cells. BM: bone marrow; ANC: absolute neutrophils count.
